# Self-Control as Conceptual Framework to Understand and Support People Who Use Drugs During Sex

**DOI:** 10.3389/fpubh.2022.894415

**Published:** 2022-06-15

**Authors:** Tom Platteau, Eric Florence, John B. F. de Wit

**Affiliations:** ^1^Department of Clinical Sciences, Institute of Tropical Medicine, Antwerp, Belgium; ^2^Faculty of Psychology, Open University, Heerlen, Netherlands; ^3^Department of Interdisciplinary Social Science, Utrecht University, Utrecht, Netherlands

**Keywords:** pharmacosex, chemsex, conceptual framework, self-control, care and support

## Abstract

Few theory-informed interventions to support people who use drugs during sex have been conceptualized and developed. We conceptualize sexualized drug use, also referred to as chemsex or pharmacosex, as a self-control challenge, and draw on extant theory and research to propose intervention approaches that can be tailored to meet the differing needs of people who engage in sexualized drug use. We draw on a continuum perspective of sexualized drug use, in particular chemsex, and discuss the role of reasoned and automatic processes in behavioral decisions, as well as critical components of effective self-control of behavior. A self-control approach can empower people to tackle their sexualized drug use, and classify their experienced sex-related drug use as problematic. Self-control encompasses clarifying one's goals and identifying strategies to mitigate behaviors to achieve these goals, despite competing pharmacosex desires. Our approach to self-control sexualized drug use contains three critical components: goal setting, goal enactment, and goal progress appraisal and goal adjustment. Goals should be formulated specific, ambitious yet realistic, and tailored to the individual's needs and wishes. Goals may target aspects of drug use, protecting sexual health and mitigating negative impacts. Implementing goal enactment implies translating goals into concrete (short-term) actions to move toward the higher-order goal via goal intentions and action/coping plans. During the goal progress appraisal and adjustment stage, people compare their actual with their planned behavior. This reflection may result in goal adjustment through feedback loops to adjust their goals and action/coping plans. We propose that our self-control approach can guide the development of interventions to effectively support people to prevent or limit pharmacosex, and helps to effectively mitigate or reduce negative impacts via self-help, peer support or professional support, offered via personal counseling or digital tools.

## Sexualized Drug Use and Potential Harms

Chemsex, “the use of drugs before or during planned sexual activity to sustain, enhance, disinhibit or facilitate the experience” (1), has become a public health concern, especially among gay, bisexual and other men who have sex with men (GBMSM) (2, 3). Chemsex is a specific form of sexualized drug use characterized by the use of potent substances [i.e., methamphetamine (“crystal meth”), mephedrone and gamma-hydroxybutyrate (GHB)/gamma-butyrolactone (GBL)] (4), albeit that differences in the specific drugs that are used have been observed between regions (5, 6) and user samples (7). Chemsex is also characterized by the contexts in which it used, notably during events that may last for several days, and its association with previously unseen harm among a specific group of GBMSM using (digital) technologies (8). People in other population groups are also found to engage in sexualized drug use, in particular swingers, heterosexual men and women who as a couple have sex with others (9). As chemsex has come to refer to specific types of sexualized drug use among GBMSM, the more inclusive term pharmacosex has been proposed to cover the range of substances that are used in conjunction with sex by diverse population groups (10). We will interchangeably use the terms sexualized drug use, and pharmacosex, unless we draw on work specifically referring to chemsex.

People initiate sexualized drug use for diverse reasons. Some people use drugs in combination with sex for hedonistic reasons, as drug use can enhance the qualities valued in sex and may increase the capability for the sex that is wanted. Others initiate sexualized drug use to increase a sense of belonging or to cope with everyday problems (11, 12). Taking drugs when having sex has in particular been found to be a coping strategy to deal with negative emotions and experiences, such as loneliness, anxiety (traumatic), stress, or low self-esteem (13). Feelings of loneliness may stem from an accumulation of intersecting adverse experiences (14).

Research has found associations between sexualized drug use, in particular chemsex, and a variety of sex and drug-specific health and social harms. Possible drug related health harms include dehydration, hyperthermia, drug-induced violence and injuries, psychosis, overdose and drug dependence (15). Furthermore, the majority of chemsex users combine different substances during a chemsex event (16–19). This polydrug use exposes the individual to even higher risks due to the combined effects (15, 20) and is associated with a higher risk of drug overdose (20). In addition to these drug related harms, chemsex is also associated with sexual risk behaviors, such as a large number of sexual partners, transactional sex, sharing sex toys, prolonged sexual sessions, condomless anal intercourse and other higher risk sexual practices, for example fisting (21–25). These behaviors increase the risk of sexually transmitted infections (STI), including HIV (21, 24, 26–28), and/or infections with (other) blood-borne viruses (e.g., hepatitis B or hepatitis C) (22). Chemsex is also associated with non-consensual or unwanted sex (6, 29). Furthermore, adverse mental health outcomes, such as anxiety and depression, have also been reported to be associated with chemsex (30, 31), especially when users inject drugs (“slamming”) (32).

## Sexualized Drug Use Prevention and Support

In the context of these potential adverse impacts, recent research finds that some GBMSM who engage in sexualized drug use experience a need for support. An online survey among 511 GBMSM attending an STI-clinic in the Netherlands showed that 23% of men who engaged in chemsex expressed a need for professional counseling (6). These chemsex users primarily sought more information about existing healthcare services and peer support. However, where such services exist, GBMSM are found to encounter barriers related to both access to services and individual service providers' attitudes (33). Research has documented a need for dedicated, non-judgmental, and possibly anonymous support among GBMSM engaging in chemsex, as they may experience shame, fear of being recognized (26, 34), and stigmatization by their healthcare provider (35).

Care and support programs for GBMSM who engage in sexualized drug use remain limited. Some peer-based initiatives have been initiated to support GBMSM through harm-reduction services, peer support, health promotion, strengthening communities, training professionals and investing in advocacy and policy (8, 34–38), as well as mindfulness and yoga (personal communication). Behavioral interventions have been implemented, making use of text-messages communication (39), expressive writing (40), safer sex counseling (41), behavioral activation (42), personalized cognitive counseling (43) and motivational interviewing and cognitive behavioral therapy (44). Pharmatherapeutical approaches have also been implemented in healthcare settings (45, 46). In addition, some interventions using digital technology are in the pipeline, including for GBMSM who use chemsex in Hong Kong (47), the United States (48) and Belgium (49). The latter is conceived as an intervention to make chemsex users more conscious about their use, with the aim of reducing and tackling negative impacts of chemsex (49).

Despite these initiatives, the absence of evidence-based interventions to support people who engage in chemsex is concerning (6, 26, 49, 50). As is well-established in health promotion planning [e.g., (51)], we propose that appropriate and effective support programs for sexualized drug use should be adapted to the needs of beneficiaries, and grounded in theory and evidence. People who engage in sexualized drug use may have a variety of support needs, including drug related issues (e.g., dependence), as well as issues related to sexual health and the relational, professional and social implications of their sexualized drug use. Support should hence be tailored to these specific needs and provide different treatment options, including to tackle various interconnecting issues. In this paper, we first provide an outline of a conceptual perspective to guide support for people who engage in sexualized drug use. Subsequently, we propose intervention approaches aligned with the conceptual framework to address pharmacosex users' diverse needs. The suggested intervention components can be integrated in dedicated and comprehensive approaches in community and healthcare settings.

## Theories to Understand Sexualized Drug Use

An important question to understand and prevent potential adverse impacts among people who engage in sexualized drug use is why people initiate and continue to engage in pharmacosex. To answer this question, we draw on a continuum perspective of chemsex use, a social cognition perspective on the interplay between reasoned and automatic processes in behavioral decisions, and critical components of effective self-control of behavior.

### Continuum Perspective of Chemsex

Most definitions, views and understandings of substance use, including chemsex, reflect a binary perspective of problematic vs. non-problematic use (52). This binary perspective has major implications for support approaches, as it suggests that only problematic use requires support, and abstinence generally is the main focus of such support [cf. (53)]. This, however, obscures the importance of preventing chemsex behavior from becoming problematic, and the need for appropriate and tailored support for people who do not (yet) experience (major) adverse impacts of sexualized drug use. Support should thus be available for all chemsex users who require it, not solely for those whose use is considered “problematic” according to some sort of standard. Rather, it is important to effectively respond to self-perceived support needs of chemsex users (35).

To tailor support to the varying needs of chemsex users, a continuum-model of chemsex use has been proposed (14). This model describes chemsex use as a journey, with problematic chemsex as a possible but not inevitable outcome (14). A critical assumption of the journey model is that even though chemsex need not be problematic, it nevertheless carries the potential for harm. The journey model describes a spiraling process of chemsex use across six “stages,” from its onset toward severe health impact caused by chemsex use. In the earlier stages, some people who engage in sexualized drug use may not experience any harm as they can accommodate drug use in their life, while other users in those same stages may experience negative impacts. It is the self-perception of adverse impacts that is critical to whether chemsex is problematic or not, and this self-perceived impact underlies demand for support. The individual's evaluation of the impact of sexualized drug use on their everyday life (e.g., relational, professional, psychological and health aspects) is thus key in the understanding of the problematic character of sexualized drug use.

### Reasoned and Automatic Processes in Sexualized Drug Use

Using drugs while understanding its risks, and possibly even experiencing adverse impacts, seems paradoxical, and in the past decades (problematic) substance use has been explained as a chronic brain disease (54, 55). From the perspective of this brain disease model of addiction (BDMA) (56, 57), a vulnerable brain gets taken over by addictive substances (58), making behavior progressively less voluntary and more compulsive. How these involuntary processes affect people's actions can be understood from the perspective of dual systems models of behavior.

Dual-systems models of decision-making and behavior distinguish between an automatic and a reasoned pathway to behavior [e.g., (59)]. The automatic pathway represents a fast, impulsive system, while the reasoned pathway reflects a slower, reasoned system. In the case of sexualized drug use, the automatic, impulsive system can be thought of as a representation of the influences stemming from a strong, compulsive, desire for the immediate experience that is enabled by sexualized drug use. The slower, reasoned system involved in sexualized drug use encompasses a person's deliberate consideration of its various positive and negative outcomes. Dual systems models suggest that behavior can result either from automatic processes or reasoned decisions, with the *BDM*A positing that continued substance use mostly reflects automatic processes. An alternative multi-systems model, however, proposes that substance use is not fully cue-driven and rigid, reflecting a loss of choice as the BDMA suggests, but results from a biased choice as drug use becomes more attractive (53). We propose that as people progress through their chemsex journey, automatic, cue-driven processes come to dominate substance use, through biases related to triggers in situations that people may frequently encounter (e.g., pharmacosex events), the formation of habits, and dependence or addiction to substances.

### Self-Control Processes in Sexualized Drug Use

People may experience their sexualized drug use as problematic if automatic processes conflict with and override the reasoned decisions they want to make. For instance, when sexualized drug use comes to be experienced as dependence and interfering with other important life goals, and as exceeding self-control. Self-control is a cognitive process that refers to one's ability to forego immediate or momentary pleasures, which may have negative consequences, in favor of longer-term, more abstract benefits or the prevention of adverse impacts that may never occur (60). In the case of sexualized drug use, self-control refers to, for instance, foregoing the immediate excitation of combining sex and drugs to avoid potential negative impacts, including physical harm, legal sanctions, social disapproval or self-disappointment. Self-control encompasses the idea that how people react to and deal with temptations in the here and now is affected by their consideration of potential outcomes (61), which is enabled and limited by the human capacity for foresight and simulation (62).

Kotabe and Hofmann provide an overview of the challenges and processes involved in effective self-control (63). Their *integrative self-control theory* posits that behavioral enactment depends on how potential conflict between disparate action tendencies is resolved. Action tendencies can result from a higher order goal, such as to protect one's health, which is associated with reasoned, reflective decisions based on a consideration of long-term benefits (64–66). Alternatively, action tendencies can also result more automatically or impulsively from a current desire, a state of wanting (67), that directs a person toward immediate, rewarding stimuli such as sex or drugs. When this desire is incompatible with the higher-order goal, a desire-goal conflict occurs. This conflict may trigger a self-control effort, if people have the capacity and motivation for self-control (63). According to the integrative self-control theory, one's behavior ultimately depends on the relative strength of the desire and the self-control effort, assuming there are no constraints to enact either the higher-order goal or the desire.

Sexualized drug use can impact self-control in two ways: directly via a strong desire resulting from sexual arousal that can conflict with higher-order goals, as well as indirectly by substance related intoxication. This intoxication impairs an individual's motivation and capacity for self-control to pursue and attain their higher-order goals. When desires related to sexualized drug use conflict with higher order goals, people may actively want and try to control their sexualized substance use. Whether their self-control efforts are successful will depend on their motivation and capacity for self-control, which is likely affected by the use of substances. If people do not succeed in this self-control of sexualized drug use, they might look for help if they experience their behavior as problematic. In our conceptualization, seeking support reflects an individual's evaluation of their situation, including relational, professional, psychological and health aspects, and gaining access to support should not depend on any external definition of problematic pharmacosex.

## Promoting Self-Control of Sexualized Drug Use

A critique of dominant perspectives of substance use and addiction, in particular the BDMA, is that this suggest that “there is no road back to controlled use or recovery” [cf. (53) p. 112]. However, it has been noted that many if not most people experiencing addiction recover (53), and the finding that most people relapse may be a misrepresentation based on overrelying on samples in addiction settings (68). We propose that effective self-control is an important leverage point for programs to appropriately support pharmacosex drug users across the sexualized substance use continuum.

Strengthening self-control can play a role in preventing initiation of use, supporting controlled use and harm reduction, as well as enabling discontinuation of use, depending on the individual's situation and wishes. Furthermore, self-control can be bolstered through various approaches, including (digital) self-help tools, peer-based support and professional care throughout the different stages of the chemsex journey. Here, we highlight the importance of approaches to support self-control that focus on enabling people to act on their (higher order) goals. This perspective complements established and controversial views of addiction control that emphasize the importance of continued abstinence to mitigate the overriding influence of substance-related desire [see (53)].

**Figure 1:**
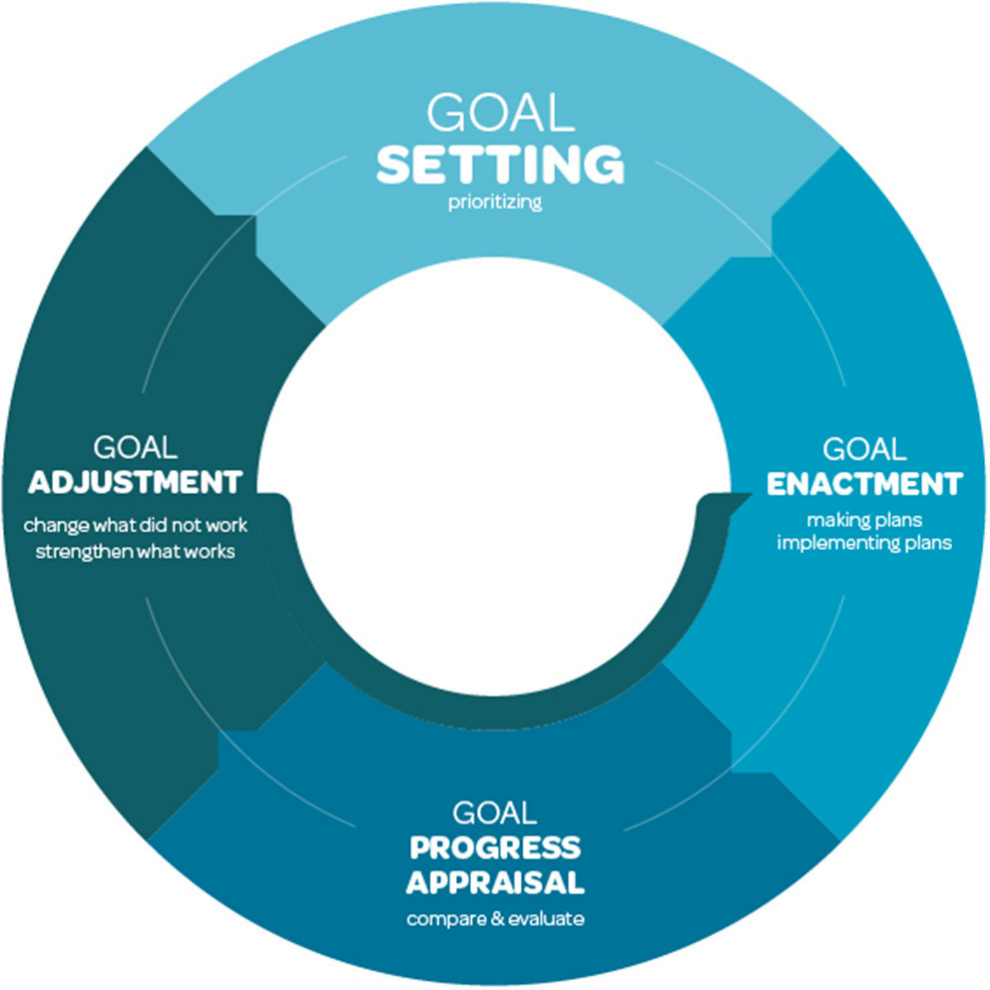
Self-control approach of sexualized drug use support.

The starting point for our self-control perspective with sexualized substance use should be to assist users in clarifying their higher order goals. Subsequently, users should be encouraged to identify strategies to prevent that behaviors to achieve their higher-order goals are undermined by competing pharmacosex-related desires. Drawing on theories of goal-directed behavior, we propose a self-control approach to support sexualized drug use that distinguishes three critical components: goal setting, goal enactment, goal progress appraisal and goal adjustment (see Figure 1).

### Goal Setting—Prioritizing

Goal setting theory (69, 70) is based on the observation that conscious human behavior is purposeful, and goal setting is an effective way of translating abstract wishes into concrete goals. Several goal characteristics have been found to affect the impact of goal setting on goal achievement. The technique of mental contrasting can help people to identify and set effective goals. When employing mental contrasting, people imagine the attainment of a desired future, and reflect on the present situation that obstructs this imagined future (71).

A goal should be specific, realistic and ambitious [e.g., (72)], and defined as a specific learning goal (73) that starts from the individual's personal situation. An ambitious goal generates more effort urging people to be committed to their goal (motivation). This commitment is reflected in the desirability and feasibility of the goal (71), where desirability refers to the importance of the goal (level of pleasantness when achieving it), and feasibility to the individual's self-efficacy and expectations (71, 72).

Applied to pharmacosex support, specific goals that people may want to achieve will differ, and goal setting will need to reflect individuals' priorities. Potential goals that require self-control in the context of sexualized drug use can, among others, be related to aspects of drug use (e.g., which substances and combinations to use/avoid, frequency of use), protecting the (sexual) health and wellbeing of oneself and others (e.g., reducing the risk of STI, ensuring consensual sex) and mitigating adverse social impacts (e.g., relationship conflicts, professional achievements). Once peoples' goals have been clarified, strategies can be put in place to optimize enactment.

### Goal Enactment—Making a Plan, and Sticking to It

Goal enactment refers to the planning and execution of specific actions to achieve the higher-order goal that is as reflected in goal intentions (e.g., “I want to limit my sexualized drug use”). A goal intention is “the instruction that people give themselves to perform particular behaviors or to achieve certain desired outcomes” (74). Although considered a critical predictor of health behavior, research shows that people render their “good” intentions into action in only 53% of the time (75). This discrepancy between peoples' intentions and behavior is typically referred to as the intention-behavior gap, reflecting that forming goal intentions is necessary but insufficient for goal attainment (76, 77).

Goal intentions typically reflect peoples' broad aspirations, and it has been suggested that these need to be accompanied by more specific plans that specify concrete actions that need to be undertaken to achieve the goal (71, 76, 78, 79). Such specific plans can take the form of implementation intentions (78, 80), which complement goal intentions and take the form of if-then plans: if a certain situation arises, then I will act like this (e.g., “If I go to the next chemsex event, then I will only use the drugs I brought myself”). The if-then format requires considering two distinct aspects of the process of goal enactment: an effective behavior to achieve one's goal, and a suitable situation to initiate this behavior. A meta-analysis has shown that implementation intentions increase the likelihood that people enact their goal intentions, with effect sizes found to be medium to large (81).

Implementation intentions can be concerned with getting started with a particular behavior (e.g., action plans), or with navigating challenges along the path of behavior change (e.g., coping plans). The purpose of an action plan is to translate a goal intention into concrete and feasible actions. In the case of pharmacosex use, an action plan can refer to the behaviors that a person plans to enact at, for instance, the next chemsex event, that contributes to achieving the predefined goal. For instance, a goal could be to have safer sex during pharmacosex, which could be guided by the goal intention “at the next pharmacosex event I will have safer sex.” A related action plan to ensure attainment of the goal intention could be “I will make sure to take PrEP to protect myself during the next pharmacosex event.” Action planning has been used to promote health behavior change in different domains [see (82)] and a recent meta-analysis has also found the forming of plans to be effective in reducing substance use (83).

Coping plans are concerned with overcoming specific barriers to (continued) action that may interfere with goal directed behavior (76), such as distractions and challenges that require effort or persistence. Effective coping planning requires experience and increases over time. In the context of pharmacosex, coping plans could, for instance, be related to preparing oneself to deal with challenging circumstances during a pharmacosex event, such as “I will set an alarm on my phone to remind me to take PrEP during the pharmacosex event” to prevent forgetting to take PrEP, for instance due to a loss of sense of time. Table 1 gives further examples of goal intentions, action plans and coping plans, related directly to the self-control of drug use, as well as the self-control of behaviors to mitigate sexual health risks and potential adverse social impacts of pharmacosex use.

**Table 1:** Examples of goal intentions, action plans and coping plans for the self-control of sexualized drug use and to mitigate potential health and social impacts.

	**Drug use**	**Sexual health**	**Social situation**
Goal intention	I only want to use my own GHB throughout the event	I want to contribute to reducing the risk of STI in my community	I want to ensure that chemsex does not interfere with my job performance
Action plans	If I plan going to a chemsex event, I will order GHB to take it with me	If I contract an STI, I will inform my sexual partners	If I plan an event on Saturday, I will have sufficient time to recover by Monday
Coping plans	To avoid running out of GHB too fast, I will keep a log of when I'm taking GHB	To avoid feelings of shame, I will make use of an online tool to notify my partners anonymously	To avoid losing track of time, I set an alarm on my phone to remind me to go home

### Goal Progress Appraisal and Adjustment—Comparing, Evaluating, and Adapting

Goal appraisal entails that people compare their behavior to the goal they had set, and evaluate whether they have achieved the goal or not. The outcome of this comparison may result in goal adjustment, reflecting feedback loops that play a critical role in self-control [see (84)]. For instance, some people intend to change their pharmacosex behavior toward a healthier alternative (e.g., not engaging in pharmacosex at all), yet find that they did not behave as planned. This experience of failure to achieve one's goal is a frequent challenge for attempts to change behavior in many health domains, and may contribute to abandoning the goal (e.g., “I am not going to succeed to stop my pharmacosex”) (76). Alternatively, failure to achieve one's goal may also constitute a learning experience for people to adjust their behavior change goal in ways that are more realistic and achievable (e.g., “I am going to engage in pharmacosex less frequently”). Furthermore, action and coping plans may be adjusted to better fit the situational challenges that people encounter (i.e., change what did not work). If, in contrast, the intended behavior has been achieved, more ambitious behavior change goals may be set (e.g., “I want to reduce my pharmacosex engagement from weekly to monthly”), and action and coping plans can be adjusted to enable the achievement of these more ambitious goals (i.e., strengthen what works).

Goal adjustment is not so much the final step in the self-control process, as it is the start of a new goal/action loop (see Figure 1). When goals and action/coping plans have been adjusted, the next loop in the iterative self-control process starts. Goal/action loops enable repeated goal adjustment and may continue until the individual reaches the point where they feel they have achieved their goal or (temporarily) abandon the goal and possibly restart a change process in the future. The process of goal appraisal and adjustment can occur following as well as during engagement in pharmacosex, and draws on processes of action control. Action control entails that a behavior is evaluated against a behavioral standard (85), which can occur during a pharmacosex event (i.e., concurrent action control), and reflecting on the implications of experiences for future events (i.e., prospective action control).

## Self-Control of Sexualized Drug Use in Practice

We propose that the successful promotion of self-control in pharmacosex need to encompass approaches to support goal setting, goal enactment, and goal progress appraisal and adjustment. Such approaches can strengthen support for pharmacosex provided by professionals or peers, as well as through self-help. In line with prevailing counseling approaches (i.e., motivational interviewing), in person and online support for individuals or small groups of people who engage in pharmacosex can contribute to setting behavioral goals for the self-control of pharmacosex, and formulating action/coping plans to achieve these goals. Furthermore, counseling also offers opportunities to support goal adjustment strategies. For instance, a person who engaged in pharmacosex may recall experiences during a pharmacosex event, including if they had a particular goal and plan, and whether they behaved accordingly. If the person behaved as planned and achieved their goal, this may result in a positive experience, contribute to a sense of self-efficacy, strengthen self-control, and, consequently, reduce the risk of harm. Moreover, this increased self-control may contribute to a positive feedback loop such that people set more challenging subsequent goals to manage their engagement in pharmacosex, and adjust their action/coping plans to achieve these goals. This process can be repeated until the person achieves their ultimate goal, which may evolve over the course of the change process.

While counseling approaches hold much promise to support the self-control of engagement in pharmacosex, this type of support is disconnected from actual pharmacosex events, and hence limited to prospective action control. Use of smartphone applications may, in addition, enable concurrent action control. Drawing on promising developments in digital health promotion, including in healthcare settings (86, 87), smartphone applications can enable real-time self-control of engagement in pharmacosex events (49).

## Discussion

In this paper, we set out a conceptual perspective to better understand pharmacosex, and guide intervention to support people who engage in pharmacosex use. Conceptual principles comprise a continuum perspective of chemsex, the altering weight of automatic and reasoned processes in behavioral decision making throughout this continuum, and self-control as a self-regulating strategy to overcome potential negative impacts of sexualized drug use. We acknowledge that this conceptual framework does not fully explain the complexity of factors that may influence people's pharmacosex use, including influential life-events (e.g., adverse childhood events, syndemics, trauma), social factors (e.g., peer norms and pressure, stigma and shame), as well as structural factors (e.g., barriers to accessing care, limited expertise of healthcare professionals) (88). Tackling these broader issues complements our primary focus on self-control capacity, and will require comprehensive programs that also include drug treatment, HIV/STI prevention and mental health care and support.

This conceptual self-control framework, as well as its translation into specific intervention components, may guide people toward improvement and self-control of their sexualized drug use, including the reduction of its negative impacts. This strengthening of one's ability for self-control can be accomplished via personal or digital support, and ideally continues after completion of an empowering support trajectory. This strengthened self-control competency can help the individual to effectively manage their pharmacosex use independently and avoid unwanted progress in their future sexualized drug use journey. This may comprise prevention of the initiation of sexualized drug use, facilitating and maintaining controlled use and harm reduction, and enabling discontinuation of use. Importantly, experiences that increase self-control competency may enable “impulsive” users to become “controlled” users, who can effectively reduce pharmacosex-related harms (31).

We propose that intervention components to support people who engage in pharmacosex can be integrated in dedicated and comprehensive approaches in community and healthcare settings, via personal support or digital self-help. In all these settings and approaches, self-control can help users to clarify their personal behavioral goals and objectives and enable them to achieve these. In order to translate the conceptual self-control perspective into practice, we point out three critical elements of the self-control process that need to be incorporated in effective interventions: goal setting, goal enactment, and goal progress appraisal and adjustment. These three elements reinforce each other via recurrent feedback loops, whereby the successful achievement of a goal can be the starting point of a next change cycle. This process continues until the individual reaches their ultimate goal, which may evolve throughout the self-control process. This self-control approach complements drug support services that tend to focus on abstinence and offers additional tools to focus on harm reduction.

## Conclusion

With this self-control framework, we aim to provide guiding principles for the improvement of prevention, and effective support and care programs and interventions. To achieve this overarching objective, specific intervention and intervention components derived from the conceptual framework should be developed and tested for their effectiveness in real-life settings. After all, the ultimate goal of this conceptual self-control framework and inferred intervention is to optimize the support and care for people who experience loss of self-control during their sexualized drug use.

## Author Contributions

TP and JW collaborated on the theoretical conceptualization and translation into practical intervention components. EF contributed by reviewing and providing valuable feedback throughout the process of development of the conceptual framework. All authors contributed to the article and approved the submitted version.

## Conflict of Interest

The authors declare that the research was conducted in the absence of any commercial or financial relationships that could be construed as a potential conflict of interest.

## Publisher's Note

All claims expressed in this article are solely those of the authors and do not necessarily represent those of their affiliated organizations, or those of the publisher, the editors and the reviewers. Any product that may be evaluated in this article, or claim that may be made by its manufacturer, is not guaranteed or endorsed by the publisher.
